# Post-Glacial Expansion and Population Genetic Divergence of Mangrove Species *Avicennia germinans* (L.) Stearn and *Rhizophora mangle* L. along the Mexican Coast

**DOI:** 10.1371/journal.pone.0093358

**Published:** 2014-04-03

**Authors:** Eduardo Sandoval-Castro, Richard S. Dodd, Rafael Riosmena-Rodríguez, Luis Manuel Enríquez-Paredes, Cristian Tovilla-Hernández, Juan Manuel López-Vivas, Bily Aguilar-May, Raquel Muñiz-Salazar

**Affiliations:** 1 Escuela de Ciencias de la Salud, Universidad Autónoma de Baja California, Ensenada, Baja California, México; 2 Facultad de Ciencias Marinas, Universidad Autónoma de Baja California, Ensenada, Baja California, México; 3 Department of Environmental Science, Policy, and Management, University of California, Berkeley, Berkeley, California, United States of America; 4 Departamento Académico de Biología Marina, Universidad Autónoma de Baja California Sur, La Paz, Baja California Sur, México; 5 El Colegio de la Frontera Sur, Tapachula, Chiapas, México; 6 Instituto Tecnológico Superior de Villa La Venta, Huamanguillo, Tabasco, México; Instituto de Higiene e Medicina Tropical, Portugal

## Abstract

Mangrove forests in the Gulf of California, Mexico represent the northernmost populations along the Pacific coast and thus they are likely to be source populations for colonization at higher latitudes as climate becomes more favorable. Today, these populations are relatively small and fragmented and prior research has indicated that they are poor in genetic diversity. Here we set out to investigate whether the low diversity in this region was a result of recent colonization, or fragmentation and genetic drift of once more extensive mangroves due to climatic changes in the recent past. By sampling the two major mangrove species, *Rhizophora mangle* and *Avicennia germinans*, along the Pacific and Atlantic coasts of Mexico, we set out to test whether concordant genetic signals could elucidate recent evolution of the ecosystem. Genetic diversity of both mangrove species showed a decreasing trend toward northern latitudes along the Pacific coast. The lowest levels of genetic diversity were found at the range limits around the Gulf of California and the outer Baja California peninsula. Lack of a strong spatial genetic structure in this area and recent northern gene flow in *A. germinans* suggest recent colonization by this species. On the other hand, lack of a signal of recent northern dispersal in *R. mangle,* despite the higher dispersal capability of this species, indicates a longer presence of populations, at least in the southern Gulf of California. We suggest that the longer history, together with higher genetic diversity of *R. mangle* at the range limits, likely provides a gene pool better able to colonize northwards under climate change than *A. germinans*.

## Introduction

Cyclical climatic oscillations during the last few million years have had a great effect on biota, causing isolation, genetic subdivision and speciation in some cases and/or hybridization and homogenization in others [1]. In many cases, the range of a species can be considered to be highly dynamic, with periods of directional or isotropic growth of range expansions, and of contractions followed by re-expansions [2]. Given that populations of temperate species suffered extirpations from glacial ice sheets, most studies have focused on them and the effects of past climate change on tropical species are still poorly understood.

Populations are dynamic in space and time, and peripheral populations are often of special conservation concern as they are predicted to experience local extinctions at a greater rate, and to have reduced recolonization potential due to smaller population sizes and more limited dispersal [3]. For tropical species, future climate change could provide suitable habitat at higher latitudes providing the opportunity for them to expand their range limits [4], [5], [6], [7]. Population expansions can leave detectable signatures in the distribution of genetic diversity [8] that help us to trace their evolution through time. Therefore, a retrospective view of demographic changes in populations can provide insights into their colonization potential under future climate change.

Mangroves are highly productive tropical ecosystems that support numerous food chains in the coastal zone and neighboring ecosystems [9]. The presence of mangroves has a strong effect on fishery yields [10], since they provide food to offshore systems as a source of carbon [11]. Mangroves also provide protection against erosion of coastlines by reducing wave energy, locking sediment in place with their roots and promoting sedimentation [12]. Therefore, understanding the response of mangrove species to climate change is of utmost importance for the management of coastal resources.

The global distribution of mangrove marsh is mainly influenced by temperature, restricting species to warm tropical and subtropical latitudes [13]. Extreme cold events have been hypothesized to explain range transformations and severe regional extinctions and latitudinal limits of mangrove distribution [8], [14], [15]. Because salt marsh ecosystems are mostly linear along coastal areas, they are likely to respond in more predictable ways to climate change than more dispersed terrestrial systems. Indeed, a recent study suggests that *Rhizophora mangle* L. experienced range limit oscillations during glacial – interglacial cycles, to the extent that modern ranges are still constrained by post-glacial re-colonization along the Brazilian coast [8].

Mexican mangroves cover 770,057 ha [16] of which 66% is located along the Atlantic coast (11% in the Gulf of Mexico and 55% on the Yucatan peninsula). Along the Pacific coast, the largest mangrove forests are located in “Marismas Nacionales”, followed by the mangrove forests along the coast of southern Mexico in Oaxaca and Chiapas. Mangroves around the Gulf of California are less extensive and they represent the northern natural limit of mangrove species along the Pacific coast [17]. Since mangrove forests of Mexico include populations at their northern range limit, one of the research questions driving our study was whether low levels of genetic diversity reported earlier by Sandoval-Castro et al. [18] for *R. mangle* in northern Mexico are a result of recent colonization or fragmentation and genetic drift of once more extensive mangroves due to climatic changes in the recent past. Recently colonized populations are expected to harbor decreased levels of genetic diversity [19], [20], [21], although exceptions to this may result when long-distance dispersal (LDD) events are increasingly frequent [22]. For temperate species that invaded new habitats following receding ice sheets, long distance dispersal events can help explain the speed of species’ advance and unexpectedly high genetic diversity in populations distant from the putative refugia [23]. However, for tropical species, climate-related advances and retreats were most likely over relatively short distances and not into vacant habitat. This is particularly true of mangroves along the east Pacific coast that are more or less linearly distributed. Although mangrove distributions are (and likely were in the past) dependent on suitable substrate and therefore are not continuous, climatic-related advances were most likely to have followed a slow diffusion model rather than LDD. Theoretical expectations of such a model are low genetic diversity and low divergence in the newly formed populations [23].

Recent colonization, or fragmentation of older populations could have important consequences as to the likelihood of these northernmost populations providing well-adapted seed sources for colonization at higher latitudes as climate changes in the future.

Therefore, we hypothesize that Gulf of California populations of Mexican mangroves are recent colonizations following climatic restriction southwards during the last glacial. Propagule sources for northward advance along the northern Pacific coast would most likely be dominated by leading edge populations in this narrow linear system. Assuming a contiguous dispersal model (local diffusion) over non-contiguous long distance dispersal, founder populations should exhibit low genetic diversity and in this stepping stone system low genetic divergence [22]; that would lead to the following expectations that we test here: 1) All mangrove species of the ecosystem should show a concordant pattern of genetic diversity; low genetic diversity in Gulf of California populations and little genetic differentiation among populations compared to those further south along the Mexican coast, 2) This pattern would be more marked along the Pacific than the Atlantic coast, because the former includes the latitudinal limit of mangroves and source populations are more linearly distributed along the Pacific than the Caribbean coast In order to elucidate these hypotheses, we analyzed and compared patterns of genetic variation of the two major mangrove species in Mexico (*A. germinans* and *R. mangle*) to interpret demographic and biogeographic processes along the Pacific and Atlantic coasts of Mexico.

## Materials and Methods

### Ethics Statement

Permission to collect samples in each location was obtained from General Directorate for Wildlife (Dirección General de Vida Silvestre de la Secretaría de Medio Ambiente y Recursos Naturales, SEMARNAT).

### Plant Material and DNA Isolation

**Figure 1:**
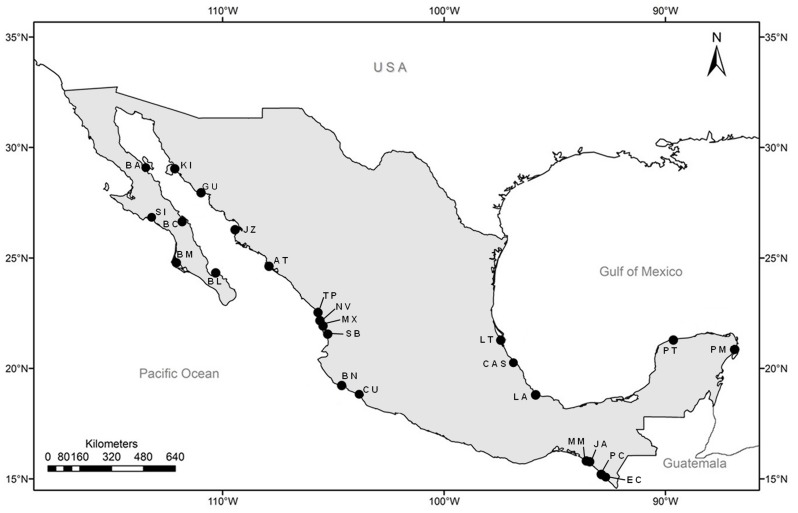
Geographic location of the sampling sites of mangrove species sampled along Mexican coasts.

Leaf tissue from 448 individuals of *A. germinans* and 600 of *R. mangle* was collected from mangrove forests along the Pacific and the Atlantic coasts of Mexico. Sampling data of *R. mangle* from the Baja California peninsula and the Gulf of California have already been reported in a previous work [18]. Remaining locations from the Central and Southern Pacific coast and from the Atlantic are new to this study (Table 1). Along the Pacific coast, sampling was performed from the Gulf of California to “La Encrucijada”, near to the Mexican border with Guatemala. Along the Atlantic coast, the sampling localities were situated in the Gulf of Mexico and around the Yucatan Peninsula (Fig. 1). Samples were taken from specimens separated by at least 30 m to prevent consanguinity and to maximize the probability of collecting diverse genotypes. The samples were dehydrated and stored in silica gel until DNA extraction. Total genomic DNA was isolated from approximately 200 mg dry weight of leaf using a modified CTAB/PVP method [18].

**Table 1:** Genetic diversity of *A. germinans* and *R. mangle* in terms of total number of alleles (A), Allelic richness (AR), expected (H_E_) and observed heterozygosity (H_O_), standard errors in parentheses. (N) Number of sampled individuals, (–) no sample.

Sampling site	Code	N	A	AR	H_E_	H_O_	N	A	AR	H_E_	H_O_
*Pacific coast*		*A. germinans*					*R. mangle*				
Bahía de los Ángeles	BA	–	–	–	–	–	†48	8	1.25	0.09	0.06
Laguna San Ignacio	SI	–	–	–	–	–	†30	9	1.43	0.13	0.09
Bahía Kino	KI	29	7	1.00	0.00	0.00	†27	9	1.33	0.10	0.08
Bahía Concepción	BC	18	10	1.29	0.08	0.07	†26	9	1.43	0.12	0.12
Bahía Magdalena	BM	34	9	1.08	0.01	0.01	†30	10	1.48	0.18	0.19
Balandra	BL	30	9	1.15	0.02	0.02	†30	11	1.60	0.18	0.26
Guaymas	GU	24	14	1.41	0.07	0.06	†28	13	1.76	0.15	0.13
El Jitzámuri	JZ	30	15	1.37	0.05	0.05	†28	12	1.87	0.26	0.26
Bahía de Altata	AT	30	19	1.94	0.21	0.21	†29	15	2.12	0.26	0.27
Teacapán	TP	30	22	2.12	0.24	0.24	†29	14	2.12	0.32	0.24
Novillero	NV	24	24	2.21	0.25	0.22	39	14	2.05	0.32	0.33
Mexcaltitán	MX	17	18	2.03	0.22	0.18	26	13	2.00	0.31	0.32
San Blas	SB	30	19	1.96	0.23	0.18	29	14	2.07	0.33	0.33
Barra Navidad	BN	10	12	1.56	0.10	0.10	10	13	2.17	0.36	0.30
Laguna Cuyutlán	CU	–	–	–	–	–	18	12	1.85	0.28	0.16
Mar Muerto	MM	16	31	3.32	0.42	0.36	–	–	–	–	–
Joaquín Amaro	JA	20	31	3.18	0.38	0.35	31	17	2.49	0.42	0.40
Laguna Panzancola	PC	10	28	3.51	0.51	0.51	30	22	2.80	0.47	0.46
La Encrucijada	EC	–	–	–	–	–	11	19	3.09	0.48	0.56
Mean Pacific				1.94(0.21)	0.19(0.04)	0.17(0.04)			1.94(0.12)	0.26(0.03)	0.25(0.03)
***Atlantic coast***											
Laguna Tamiahua	LT	28	41	3.52	0.43	0.36	30	13	1.92	0.26	0.20
Laguna Casitas	CA	–	–	–	–	–	16	12	1.99	0.25	0.27
Laguna Alvarado	LA	30	42	3.62	0.45	0.41	–	–	–	–	
Progreso-Telchac	PT	30	42	4.14	0.56	0.48	27	13	2.08	0.35	0.27
Puerto Morelos	PM	8	23	3.26	0.55	0.44	28	16	2.37	0.38	0.31
Mean Atlantic				3.64(0.18)	0.50(0.03)	0.42(0.03)			2.09(0.10)	0.31(0.03)	0.26(0.02)
**Total**		448	93	2.3(0.24)	0.26(0.04)	0.23(0.04)	600	34	1.97(0.1)	0.27(0.02)	0.25(0.03)
**Mean Pacific/Mean Atlantic**				0.53 (0.06)††	0.37 (0.08)††	0.41 (0.09)††			0.93 (0.05)††	0.85 (0.09)††	0.96 (0.09)††

† Data previously reported by Sandoval-Castro et al. [18].

†† Standard errors estimated after 5,000 bootstraps.

### Microsatellite Analysis

Individuals were genotyped at seven loci (AgT4, AgT7, AgT8, AgT9, AgD6, AgD13 and CA_002) previously designed for *A. germinans* [24], [25], [20] and six loci (Rm7, Rm11, Rm19, Rm21, Rm38, and Rm46) previously designed for *R. mangle* [26]. These repeat motifs were isolated from genomic DNA and are expected, *a priori*, to be neutral with respect to natural selection. The forward primers were fluorescent-labeled with FAM, VIC, PET and NED (Applied Biosystems Inc). All amplifications were performed on a MyCycler BIORAD thermal cycler in 20 μL PCR reactions containing 1×Buffer (10 mM Tris HCl, 50 mM KCl, pH 8.3, SIGMA), 2.0 mM MgCl_2_, 200 μM of each dNTP, 0.15 μM of each primer, 1 unit *Taq* DNA polymerase (SIGMA) and 20 ng of genomic DNA. With the exception of some small variations on annealing temperatures, all microsatellite loci were amplified with a similar thermocycler profile. The profile consisted of an initial denaturation step at 95°C for 5 min, followed by 35 amplification cycles as follows: 95°C for 30 s, annealing temperature (50°C for all the loci in *R. mangle* and for the loci AgT4, AgT7, AgT8 and AgT9, 55°C for AgD13 and CA_002 and 59°C for AgD6 in *A. germinans*), for 30 s and 45 s at 72°C, ending with an extension cycle at 72°C for 30 min. To ensure reproducibility and consistency in PCR amplification, approximately 5% of samples were re-amplified. In addition, a negative control was run for each set of PCR reactions and genotyped to check for contamination. Amplified products were run on an ABI 310 automated DNA sequencer, and microsatellite alleles were visualized and scored in the program GeneMarker 1.97 (Softgenetics).

### Data Analysis

#### Genetic diversity

To determine spatial patterns in genetic diversity that might confirm latitudinal trends consistent with recent colonization northwards, we estimated the number of alleles (A), unbiased expected heterozygosity (H_E_) and observed heterozygosity (H_O_) for each locus across all localities using GDA 1.1 [27]. Allelic richness per locus (AR) and per population were calculated using FSTAT 2.9.3 [28]. Global tests for deviation from Hardy-Weinberg equilibrium were performed using a Markov chain algorithm and linkage between all pairs of loci was estimated using GENEPOP 4.0 [29], [30] with significance levels determined using the Markov chain method. For all Markov chain tests, the default parameters in GENEPOP were used with 100 batches of 1000 iterations each. Null alleles, large allele dropout and stutter peaks were explored using Micro-Checker 2.2.3 [31].

#### Clustering analyses

We applied the Bayesian clustering algorithm implemented in STRUCTURE v2.2.3 [32] as an exploratory analysis to infer population genetic structure, assigning individuals (probabilistically) without *a priori* knowledge of population boundaries. STRUCTURE uses individual multilocus genotype data to cluster individuals into K groups while minimizing Hardy-Weinberg disequilibrium and gametic phase disequilibrium between loci within groups [32]. STRUCTURE runs were based on 500,000 iterations after a burn-in of length 500,000 and assumed correlated allele frequencies and an admixture model with an estimated proportion α of admixed individuals. To check for Markov chain Monte Carlo (MCMC) convergence, we performed 10 replicates for each K value and checked the consistency of results. The most likely number of clusters (K) was considered to be the K value with the highest Pr(X|K) [32], [33]. Also, the optimal K value was calculated after the ΔK method described by Evanno et al. [34].

#### Genetic structure

Genetic differentiation among populations was evaluated at each locus and over all loci by calculating the measures of relative genetic differentiation among populations defined under the infinite allele model (IAM; F_ST_) [35] and the stepwise mutation model (SMM; R_ST_) [36], [37]. The presence of phylogeographic structure was assessed by using SPAGeDi 1.4 [38] through permutations of allele sizes among alleles within a single locus (*p*R_ST_) (10,000 permutations). This analysis compares the observed R_ST_ value (before randomization) with the distribution of *p*R_ST_ values obtained for all possible configurations of allele size permutations. If observed R_ST_ is within the upper 5% of the distribution of *p*R_ST_ the contribution of mutations to population differentiation is non-negligible compared with genetic drift and migration [39]. Population genetic structure was also examined using hierarchical analysis of molecular variance (AMOVA) among the clusters determined by STRUCTURE v.2.3.3. At this level, genetic differentiation was quantified with F-statistics [35] using ARLEQUIN v.3.5. [40]. The distribution of genetic variation was assessed at four hierarchical levels: among groups, among populations within groups, among individuals within populations and within individuals [41]. Statistical significance of the variance was tested by 10,000 non-parametric permutations.

#### Migration rates

On the basis of Structure and SPAGeDi analysis, we classified localities into populations. The magnitude and direction of gene flow were estimated among populations for which mutations had a non-significant effect on differentiation R_ST_≤*p*R_ST_. Estimates of evolutionary patterns of gene flow were obtained according to the maximum likelihood approach implemented in MIGRATE v.3.2.7 [42], [43]. MIGRATE uses a coalescence approach to estimate migration rates (Nm) among populations, assuming a constant per-locus mutation rate. This approach is judged to estimate gene flow more accurately than other F_ST_ methods, especially when multiple loci are employed [42]. We used 10 short-chain searches and three long-chain searches over the number of assayed microsatellite loci to obtain the magnitude and direction of gene flow according to Beerli and Felsenstein [42]. For each locus, the program was run for 10 consecutive exploratory chains with lengths of 5×10^6^ genealogy visits to adjust the driving values for both the run and the 3 long chains; the last chain was used to generate the presented results. Each of the long chains visited 5×10^8^ genealogies, sampling 5000 after an initial burn-in of 10000 steps. The program assumes discrete populations and generations, mutation-drift equilibrium, non-selective effects and the stepwise mutation model for microsatellite markers [44]. Furthermore, we estimate recent levels of gene flow between populations by using the program BAYESASS [45], which uses transient levels of linkage disequilibrium produced by recent migrants or their immediate descendants to infer levels of migration into populations. The program uses MCMC sampling in a Bayesian statistical framework to estimate gene flow in the recent past. The run involved 3×10^6^ MCMC iterations, discarding the first 10^6^. The program estimates the mean value for migration rate, and a 95% confidence interval for the estimate.

#### Isolation by distance

To test the significance of Isolation By Distance (IBD), the correlation between genetic and geographic distance matrices was tested using a Mantel test with 2000 permutations [46]. The geographical distances between samples were based on coastline distances and the genetic distance was expressed as F_ST_/(1−F_ST_) following Rousset [47]. IBD analysis was only performed on the mainland Pacific coast populations (KI, GU, JZ, AT, TP, NV, MX, SB, BN, CU, MM, JA, PC and EC). Given that mangroves from Atlantic (LT, CAS, LA, TP, PM) do not have a clear connection with the Pacific they were removed from this analysis. Because of potentially complex scenarios of dispersal into and around the Gulf of California, populations SI, BM, BL, BC, and BA were not included in the IBD analyses. Since we hypothesized a recent colonization of northern mangrove populations, we also performed an IBD analysis restricted to the northwestern coast (KI, GU, JZ, AT, TP, NV, MX, SB).

## Results

### Genetic Diversity

Microchecker analyses showed no evidence of null alleles nor allelic drop out that might interfere with posterior analysis on genetic diversity. We detected 93 alleles in *A. germinans,* of which 35.5% were exclusive to the Atlantic, 35.5% to the Pacific and 29% were common to both coasts. For *R. mangle,* only 34 alleles were detected, of which 11.8% were exclusive to the Atlantic, 38.2% to the Pacific and 50% were shared between both coasts.

**Figure 2:**
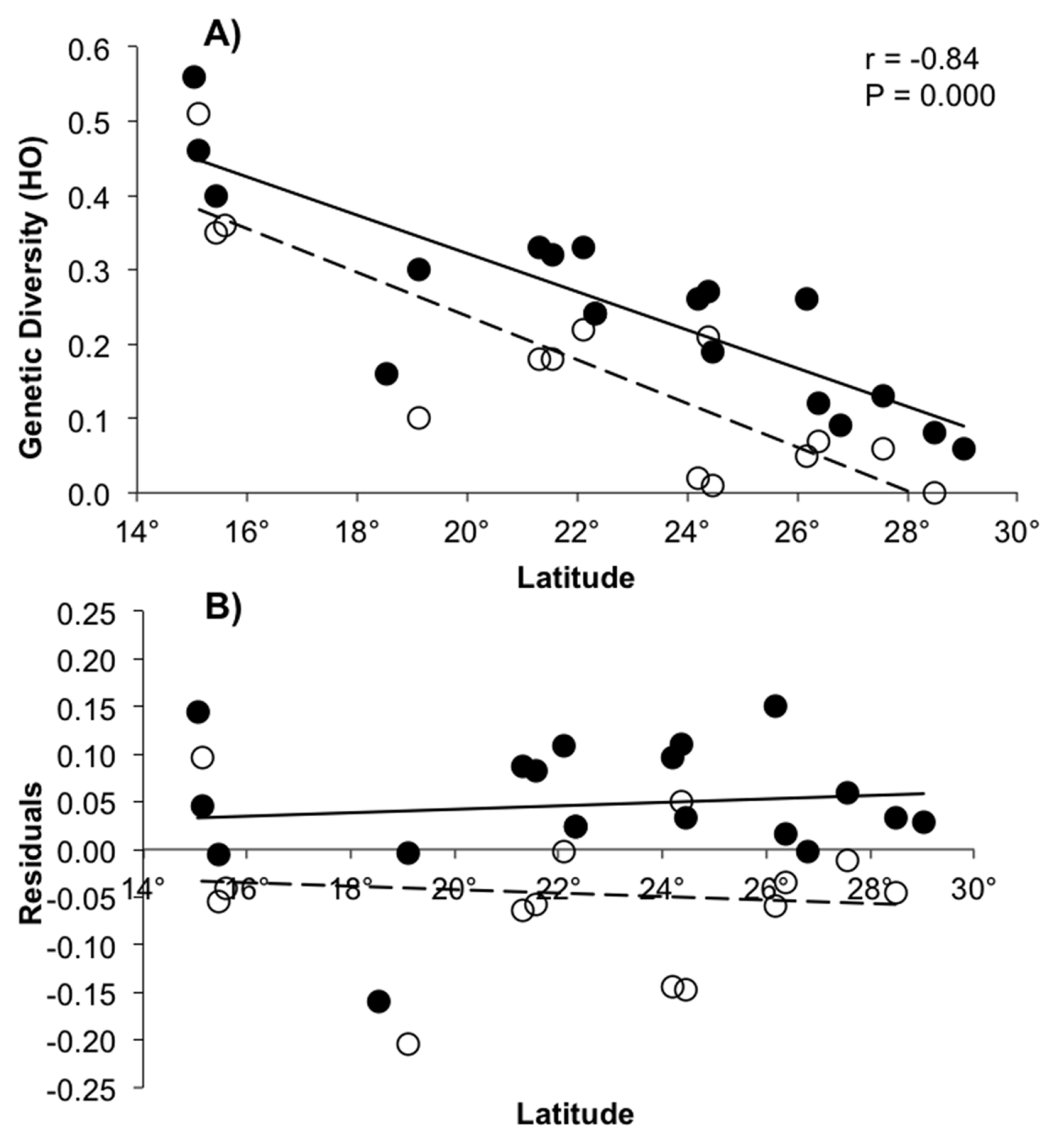
Lower genetic diversity was observed at northern margin limits. A) Genetic diversity decreases with latitude in mangrove species *A. germinans* (open circles, dashed line) and *R. mangle* (black circles, solid line) along Mexican Pacific coast. (r = Pearson’s correlation). B) ANCOVA analysis showing lower genetic diversity of *A. germinans* at Pacific coast (α = 0.05, P = 0.001).

Genotypic linkage disequilibrium was not detected among any of the pairwise loci comparisons across all populations. This suggests no evidence for selective sweeps and supports the expectation that the markers are neutral with respect to natural selection. Both mangrove species showed lower levels of genetic diversity around the Gulf of California, where mangrove forests are less extensive and more fragmented. Over all populations, genetic diversity was not significantly different between *A. germinans* and *R. mangle* (Table 1). For *A. germinans*, we detected higher levels of genetic diversity in the Atlantic populations, whereas for *R. mangle* greater diversity was detected among Pacific coast populations. Since direct comparisons of diversity at the different loci for the two species cannot be made, we standardized genetic diversity along the Pacific coast to that of the Atlantic coast by dividing mean Pacific diversity by mean Atlantic diversity (Table 1). Genetic diversity in both mangrove species showed a decreasing trend northward along the Pacific coast; lowest values were detected along the northern coasts of the Gulf of California, where mangrove species reach their natural range limits (Pearson correlation coefficients between observed heterozygosity and latitude r = −0.84 P<0.0001 for both *A. germinans* and *R. mangle* (Fig. 2A). For all three measures of genetic diversity, a ratio close to one for *R. mangle* indicated no significant difference in genetic diversity between the two coasts. However, for *A. germinans*, ratios ranged from about 0.4 to 0.5, indicating much lower diversity along the Pacific coast. This lower genetic diversity was supported by an ANCOVA analysis applied to the corrected variation (residuals) of genetic diversity (HO) versus latitude as a covariate (P = 0.001; Fig. 2B).

### Clustering Analyses

**Figure 3:**
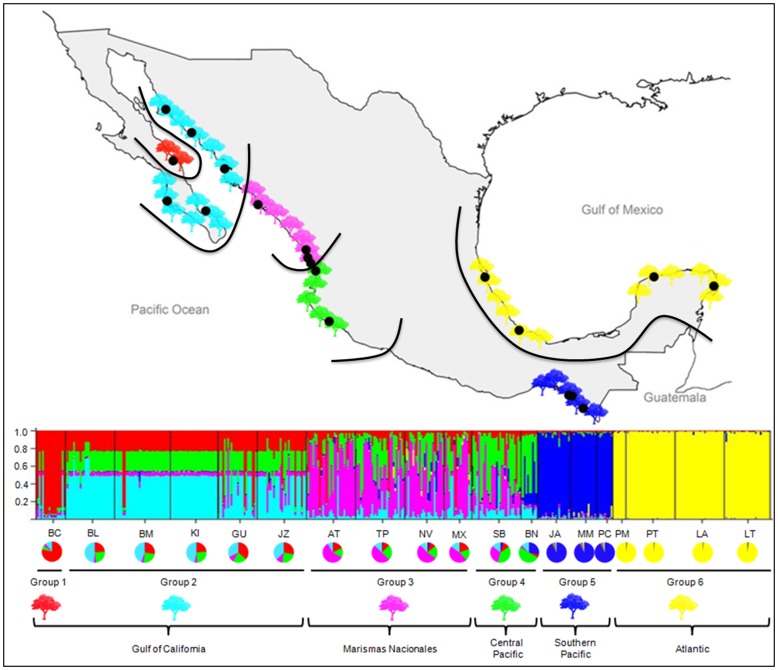
STRUCTURE plot of *Avicennia germinans* along Mexican coasts. Lines in the map indicate the spatial borders of the groups; vertical bars represent individuals whose genotypes have been apportioned into 6 clusters; colors in pie charts represent the percentage of assignment (Q values) of each group.

**Figure 4:**
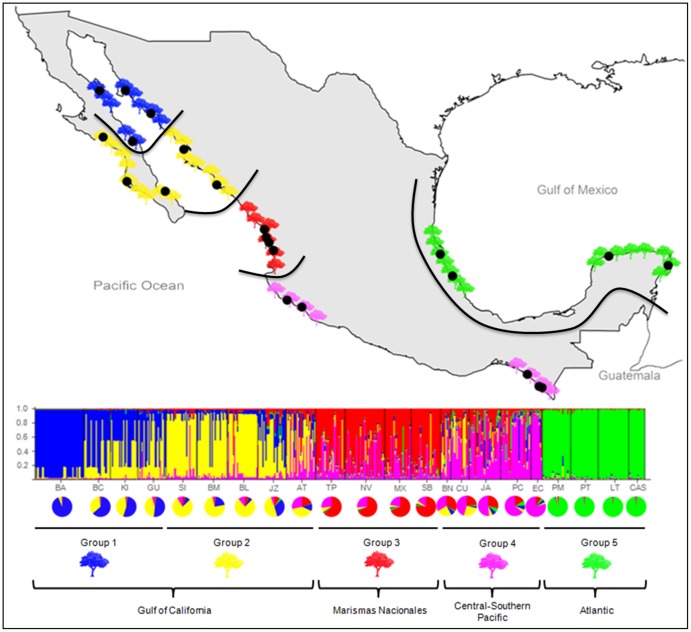
STRUCTURE plot of *Rhizophora mangle* along Mexican coasts. Lines in the map indicate the spatial borders of the groups; vertical bars represent individuals whose genotypes have been apportioned into 5 clusters; colors in pie charts represent the percentage of assignment (Q values) of each group.

Applying the Bayesian analysis in STRUCTURE and the approach of Evanno et al. [34], the most likely number of genetic clusters was six for *A. germinans* and five for *R. mangle*. Of the six clusters for *A. germinans*, five were detected along the Pacific coast and one along the Atlantic coast (Fig. 3). Although proportional assignment of individuals to clusters indicated admixture in most populations, the admixture proportions of the STRUCTURE clusters revealed five spatially distinct groups (shown in lower frame of Fig. 3). Gulf of California and peninsula populations formed, two groups; one more or less homogeneous group including populations BL, BM, KI, GU and JZ, and a group comprising a single sampling location (BC) shown in red in Fig. 3. The latter showed very little admixture of the STRUCTURE clusters and was most likely the result of a recent founder event by an individual with a genotype that was unrepresentative of the nearby populations that were likely the colonizing source. Sample locations (AT, TP, NV, MX) from “Marismas Nacionales” formed a third group that graded into the Central Pacific coast group (SB, BN). However, these sampling locations shared genotypes among themselves and with those from the Gulf of California. The fifth group included southern sampling locations along the Pacific coast (MM, JA, PC) and comprised a single STRUCTURE cluster, with individuals having minimal levels of admixture Although a spatial pattern was evident for *R. mangle*, only the Atlantic coast sampling sites formed a unique cluster (Fig. 4). Pacific coast populations formed four clusters with high levels of admixture at all but the most northerly sampling locations.

### Genetic Structure

In general, overall pairwise values of F_ST_ were lower than R_ST_, but significantly different from zero. AMOVA analysis showed that most of the genetic variation was among clusters defined by STRUCTURE (Table 2). According to SPAGeDi analyses, differentiation among all populations taking into account allele sizes (R_ST_ = 0.60 and 0.80 for *R. mangle* and *A. germinans* respectively) was significantly larger (**α = **0.05, P<0.0001) than differentiation based on allele identities (F_ST_ = 0.47 and 0.54), indicating that stepwise mutations contributed to overall among-population differentiation. However, when we only analyzed populations from the northwestern coast (“Marismas Nacionales” and “Gulf of California”), in both mangrove species R_ST_ was not significantly higher than F_ST_ (**α = **0.05, P = 0.379), suggesting that more recent evolutionary processes of migration and genetic drift were responsible for differentiation over this geographic range.

**Table 2:** Population genetic structure of *A. germinans* and *R. mangle* in Mexico.

	*A. germinans*						*R. mangle*					
	IAM			SMM			IAM			SMM		
Source of variation	% var.	F_ST_	P-value	% var.	R_ST_	P-value	_% Var._	F_ST_	P-value	_% Var._	R_ST_	P-value
Among groups	52.95	0.52*	0.000	81.00	0.81*	_0.000_	_44.82*_	_0.45*_	_0.000_	_58.50*_	_0.58*_	_0.000_
Among populations within groups	5.67	0.12*	0.000	2.35	0.12*	_0.000_	_6.65*_	_0.12*_	_0.000_	_5.46*_	_0.13*_	_0.000_
Among individuals within populations	4.33	0.10*	0.000	2.79	0.16*	_0.000_	_3.22*_	_0.06*_	_0.000_	_0.00_	_0.00_	_0.854_
Among all individuals	37.05	0.62*	0.000	13.86	0.85*	_0.000_	_45.31*_	_0.54*_	_0.000_	_33.37*_	_0.62*_	_0.000_

Hierarchical AMOVA calculated by the Infinite Allele Model (IAM) and the Stepwise Mutation Model (SMM); percentage of molecular variation (% var.) explained by the hierarchical level. *Statistically significant (p<0.05). The group numbers and population assignment was according to STRUCTURE results.

### Isolation by Distance

**Figure 5:**
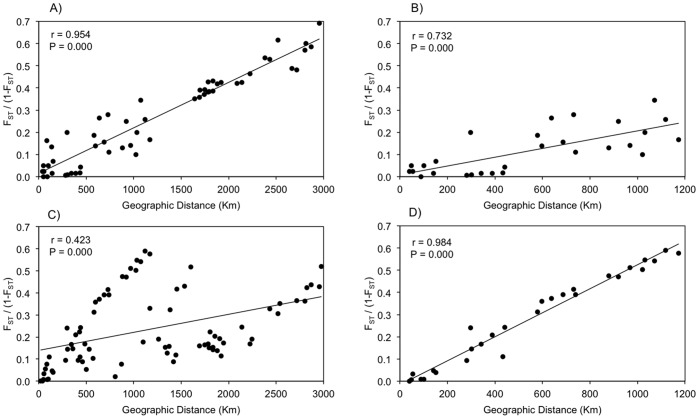
Isolation by distance. Correlations and probabilities were estimated from a Mantel test with 2000 repeats of bootstrap resampling. The y-axis is F_ST_/(1−F_ST_) following Rousset (1997). A, B) *A. germinans,* C, D) *R. mangle* on Pacific and northwestern coast, respectively.

For *A. germinans*, isolation by distance was significant over all sampling locations along the Pacific coast and over the more restricted geographic range of the northwestern coast (Fig. 5A, B). For *R. mangle*, isolation by distance was significant only over the more restricted northwester geographic range (Fig. 5C, D).

### Migration Rates

**Figure 6:**
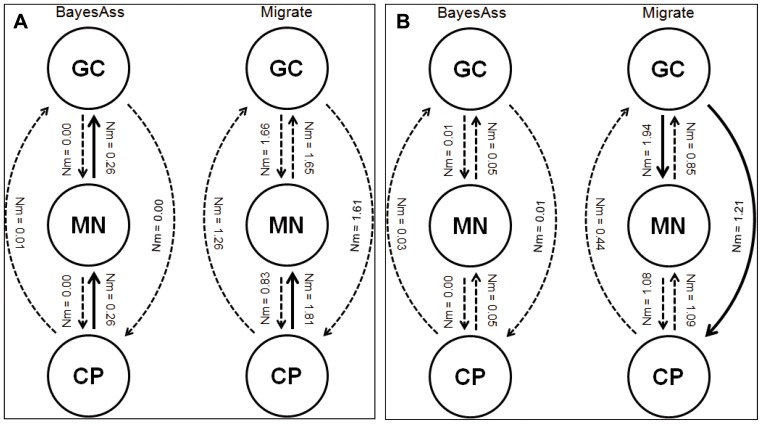
Estimates of the historical gene flow and migration directions among Gulf of California (GC), Marismas Nacionales (MN) and Central Pacific (CP). Numbers and arrows performed by MIGRATE; and estimates of recent migration rates and directions performed by BAYESASS for the northwestern Mexican mangrove populations of A) *A. germinans* and B) *R. mangle*. Solid arrows show significant gene flow.

In view of the minimal effect of mutations on genetic structure along the north-west coast, we focused on estimating directional gene flow between the Gulf of California (GC) and “Marismas Nacionales” (MN), including Central Pacific (CP) as a control (Fig. 6). Evolutionary-scale rates of migration (MIGRATE) showed a higher magnitude than contemporary-scale rates (BAYESASS). In *A. germinans* the estimated number of migrants per generation (Nm) ranged from 0.83 to 1.81 and from 0.00 to 0.26 (MIGRATE and BAYESASS respectively) (Fig. 6A), whereas in *R. mangle* Nm ranged from 0.44 to 1.94 and from 0.00 to 0.05 (Fig. 6B). Bayesian estimates based on MCMC simulations performed using BAYESASS suggest that for *A. germinans* contemporary gene flow is predominantly northward along the northwest Pacific coast, with higher rates from CP to MN and from MN to GC. On other hand, for *R. mangle* the BAYESASS analysis showed no significant evidence of recent gene flow, whereas on an evolutionary-scale, MIGRATE detected greater southward gene flow from GC to MN and CP. These inferred migration rates suggest that *R. mangle* populations in the Gulf of California are older than those of *A. germinans*. This is consistent with the present more northerly distribution of *R. mangle* along the Peninsula of Baja California today.

## Discussion

Today, mangroves in the Gulf of California and along the Pacific coasts of the Baja California peninsula form the northern limit of this ecosystem in the eastern Pacific. Populations are relatively small and fragmented and prior research has indicated that they are poor in genetic diversity [18], [48], [49]. We set out to examine patterns of genetic diversity that would help determine whether low diversity in this region was a result of recent founder events, or of fragmentation and genetic drift of once more extensive mangroves due to climatic changes in the recent past. Here, we discuss our hypotheses for recent colonization in the Gulf of California in the light of genetic diversity along the Pacific and Atlantic coasts of Mexico and evaluate the roles of differential dispersal in the two major taxa of the mangrove ecosystem.

### Are Patterns of Genetic Diversity Concordant in the Two Major Mangrove Species?

We found that broad patterns of genetic diversity along the Mexican Pacific coast were consistent for both species; a linear decrease in genetic diversity with latitude, becoming impoverished in the Gulf of California. Although patterns of genetic diversity were broadly similar, demographic processes inferred from population structure and directional migration rates revealed a more complex evolution of populations and the ecosystem. For both species, populations from “Marismas Nacionales” and the Central Pacific were highly admixed and levels of admixture declined into the Gulf of California (Fig. 3, 4). Based on cluster assignments from the program STRUCTURE, we detected shared ancestry between populations of *A. germinans* from the Gulf of California and those from “Marismas Nacionales” and Central Pacific, consistent with Gulf of California populations being the product of recent founder events, for example the BC population represented by the red color in the STRUCTURE analysis. For *A. germinans,* this hypothesis was supported by estimates of recent directional migration inferred using the program BAYESASS, which showed a predominantly northward migration from “Marismas Nacionales” to the Gulf of California.

Despite comparable latitudinal trends in genetic diversity, we found contrasting demographic signals in the two species. Gulf of California populations of *R. mangle* shared very little recent ancestry with those of “Marismas Nacionales” and Central Pacific and we found no detectable recent migration among these populations. This would seem to indicate: 1) A longer presence of *R. mangle* in the Gulf of California, 2) Confounding due to unsampled source populations, 3) Effects associated with enhanced dispersability of *R. mangle* propagules. We were careful to sample most populations along the Pacific mainland coast and, although extinct populations could have been the source of Gulf populations, it seems unlikely that they would have been genetically distinct from “Marismas Nacionales” and Central Pacific. Today “Marismas Nacionales” is one of the more extensive areas of mangrove forests along the Mexican Pacific coast. Therefore, we find it unlikely that unsampled source populations will have confounded our results. Our data do indicate that *R. mangle* propagules are more effectively dispersed than those of *A. germinans*. Whereas, southern Mexican Pacific populations of *A. germinans* formed a discrete cluster in the STRUCTURE analysis, those of *R. mangle* were admixed with “Marismas Nacionales” and Central Pacific populations. The intervening coastline between central and southern populations is not suitable for mangrove establishment, so long-distance dispersal appears to have been more effective for *R. mangle*. Nevertheless, there is no evidence of shared ancestry between Gulf of California populations of *R. mangle* and those from the southern Mexican coast to suggest a source of founder propagules for Gulf populations. We find the most likely explanation of the contrasting demographic patterns between *A. germinans* and *R. mangle* is that populations of the latter species have been present somewhere in the Gulf of California longer than *A. germinans.* Interestingly, evolutionary rates of migration estimated from MIGRATE, also yielded contrasting results; no significant rates of migration between the Gulf of California and “Marismas Nacionales” or Central Pacific for *A. germinans*, but significant southward migration from the Gulf to “Marismas Nacionales” for *R. mangle.* Taken together, these data support a scenario of glacial restriction followed by Holocene latitudinal advance for *A. germinans*, and possible persistence of populations of *R. mangle* somewhere in the Gulf of California during the harsh conditions of the last glacial maximum.

### Are Latitudinal Patterns of Genetic Diversity Comparable between Atlantic and Pacific Coasts in Mexico?

Whereas, we detected a clear linear trend of decreasing genetic diversity among populations of both species along the Pacific coast, no trend was detected along the Atlantic coast. Indeed, STRUCTURE assigned all individuals from the Atlantic to single clusters for each species. These opposing trends support our hypothesis that a stronger pattern of decreasing genetic diversity would be expected along the Pacific coast because of the more linear distribution of mangroves and inclusion of the populations from the range limit here. Sherrod and McMillan [50] argued that populations of *A. germinans* along the mainland of the Gulf of Mexico advanced and went extinct during climatic cycles of the Pleistocene. Today, whereas both species are common components of Floridan mangroves, *A. germinans* extends further north than *R. mangle*. However, along the Pacific coast, *R. mangle* has the northernmost distribution [17]. We found that mean genetic diversity of *A. germinans* was about twice as great in the Atlantic compared with the Pacific coast, but that mean genetic diversity was about equal for *R. mangle*. This is in part, due to greater sharing of alleles between the two coasts in *R. mangle*; *R. mangle* shared almost 50% of alleles between Atlantic and Pacific populations, while *A. germinans* shared around 29% of alleles. This could be an effect of higher mutation rates at the studied loci in *A. germinans,* or of more extensive early dispersal of *R. mangle* before closure of the Central American Isthmus (CAI). Genetic structure analysis showed the highest genetic differentiation among populations from the Atlantic and the Pacific coasts, supporting previously reported molecular data [48], [51], [52], [53], [54], [55], [56] and highlighting the effect of geographical isolation following elevation of the CAI around 3.5 million years ago.

### Is there Evidence of Earlier Colonization by R. Mangle, a Species with More Effective Propagule Dispersal?

Today, *R. mangle* extends further north than *A. germinans* along the Pacific coast. As discussed earlier, the patterns of genetic diversity that we observed suggest a longer presence of *R. mangle* in the Gulf of California and the Baja California peninsula. This could be explained by earlier colonization of *R. mangle* because of its more effective dispersal capabilities [57], [58] and could explain the higher admixture in *R. mangle*, however, this would not explain the presence of genetic structure in both mangrove species. Therefore, we prefer an interpretation that populations of *R. mangle* persisted, perhaps in the lower Gulf, through the last glacial, which could explain the greater genetic diversity observed in *R. mangle* than *A. germinans* along the Gulf of California. However, it is difficult to explain why *R. mangle* has been more successful than *A. germinans* at the range limit along the north-western coast of Mexico. The answer may lie with shoreline topography, which may propitiate local extinction of mangroves on coastlines with no gradual gradient [59]. Climatic niche modeling for Pacific coast populations of *A. germinans* hindsighted onto last glacial maximum climate from the PMIP database, suggests that mangroves could have survived along the Sinaloa coastline and into the lower Gulf of California through the last glacial (Dodd unpublished data). This is also supported by fossil record of prop roots of A. germinans in BC population dating from the Pliocene [60]. Mangrove forests along the Gulf of California are discontinuously distributed as a narrow band along the littoral because of the persistence of rocky coastline with reduced tidal range. This condition could favor *R. mangle* over *A. germinans* because the former is more tolerant to changes in the hydroperiod, which have occurred periodically through time [59].

In conclusion, our data provide compelling evidence that the Gulf of California and outer Baja California were recently colonized by *A. germinans* through northward propagule dispersal, perhaps from relictual stands around present-day “Marismas Nacionales”. On the other hand, *R. mangle* showed no signal of recent northern dispersal despite the greater dispersal capability of this species, suggesting the presence of populations at least in the southern Gulf of California for a longer time. Future climate change could favor colonization at higher latitudes, but the source propagules must be well adapted to the arid and highly saline conditions. Populations with a longer history at a site are likely to be better adapted to local conditions than founders from populations under more benign conditions. Under this scenario, *R. mangle* would serve as a better adapted gene pool for latitudinal advances under climate change, even though *A. germinans* showed evidence of more successful northward migration in recent time.
